# Curcumin and Acute Myeloid Leukemia: Synergistic Effects with Targeted Therapy

**DOI:** 10.3390/ijms26199700

**Published:** 2025-10-05

**Authors:** Rita Badagliacca, Manlio Fazio, Fabio Stagno, Giuseppe Mirabile, Demetrio Gerace, Alessandro Allegra

**Affiliations:** Hematology Unit, Department of Human Pathology in Adulthood and Childhood “Gaetano Barresi”, University of Messina, Via Consolare Valeria, 98125 Messina, Italy; rita.badagliacca@studenti.unime.it (R.B.); manliofazio@hotmail.it (M.F.); fabio.stagno@unime.it (F.S.); giuseppe.mirabile@polime.it (G.M.); demetriogabriele.gerace@polime.it (D.G.)

**Keywords:** curcumin, acute myeloid leukemia, combination therapies, synergistic effect, apoptosis, oxidative stress, chemoresistance, microbiota

## Abstract

Acute myeloid leukemia is characterized by the presence of malignant cells and their uncontrolled growth in bone marrow. Recent studies have been focused on the ability of curcumin, a polyphenol derived from the Curcuma longa plant. The role of curcumin is currently under investigation, due to its antitumor properties and action on several pathways, including Nuclear Factor kappa-light-chain-enhancer of activated B cells, Signal Transducer and Activator of Transcription 3, Phosphatidylinositol 3-kinase/protein kinase B, and mitogen-activated protein kinase. The aim of this review is to demonstrate the possible anti-leukemic effect of curcumin, thus its ability to induce apoptosis, inhibit cell proliferation, and modulate angiogenesis. Nowadays, although multiple synergistic effects have been observed and curcumin’s efficacy has been demonstrated through several in vivo and in vitro studies, further broad and exhaustive scientific research is needed to confirm the considerable results. In fact, the low bioavailability of curcumin has limited its clinical applications, a challenge that is currently being addressed through the development of nanoformulations to enhance its stability and absorption within the body. In conclusion, curcumin exhibits antitumor properties with a favorable profile, suggesting its potential as a supportive adjunct in the treatment of patients with acute myeloid leukemia.

## 1. Introduction

### General Considerations on Acute Myeloid Leukemia

Acute myeloid leukemia (AML) is a hematological malignancy characterized by impaired hematopoiesis due to the uncontrolled, clonal proliferation and abnormal differentiation of immature myeloid blasts in bone marrow and peripheral blood. The 2022 World Health Organization (WHO) classification categorizes AML based on differentiation stages, genetic abnormalities, and prior therapies, while the 2022 European LeukemiaNet (ELN) risk stratification focuses on genetic markers to classify patients into favorable, intermediate, or adverse risk groups [1,2].

Although there has been progress in treatment, because of some specific genetic mutations or resistance to therapies, in recent years, ongoing clinical trials are identifying new treatment strategies.

The standard chemotherapy “7 + 3” regimen for AML patients consists of the administration of cytarabine for 7 days combined with anthracycline (daunorubicin or idarubicin) for 3 days. In the case of successful induction, consolidation options can be taken into consideration such as high-dose cytarabine or allogeneic hematopoietic stem cell transplantation. Moreover, FLT3 inhibitors (midostaurin, gilteritinib), IDH1/2 inhibitors (ivosidenib, enasidenib), and BCL-2 inhibitors (venetoclax) have significantly improved outcomes in specific AML subgroups [3,4].

Despite this, the mortality rate remains high and new therapeutic strategies appear to be appropriate and play a crucial role. Among these, the polyphenol curcumin has shown significant potential effects.

## 2. Curcumin: A Multidimensional Agent

### 2.1. Overview of Curcumin

Curcumin is a hydrophobic polyphenolic compound diferuloylmethane and the principal bioactive constituent of the rhizome of *Curcuma longa* (turmeric). It belongs to the class of curcuminoids, which also include demethoxycurcumin and bisdemethoxycurcumin. According to the chemical structure, curcumin has a diarylheptanoid structure, consisting of two aromatic ring systems containing o-methoxy phenolic groups linked by a seven-carbon chain with conjugated double bonds and a β-diketone moiety. This structure allows it to interact with a wide range of molecular targets, modulating transcription factors, cytokines, enzymes, and cell signaling pathways.

With its characteristic golden-yellow pigment of turmeric, this polyphenol is well known for its wide spectrum of biological properties, among these, significant anti-inflammatory, wound-healing properties, antioxidant, and antimicrobial activities, but its most notable potential is in its antitumor effects. Curcumin’s ability to modulate several signaling pathways plays a key role in the following processes: cell proliferation, apoptosis, metastasis, and drug resistance [5,6]. Specifically, curcumin has been shown to impact pathways like Nuclear Factor kappa-light-chain-enhancer of activated B cells (NF-κB), STAT3, PI3K/Akt/mTOR, mitogen-activated protein kinase (MAPK), and JAK/STAT, making it a highly versatile compound in the field of cancer research [7,8].

Curcumin also plays a significant role in primary and secondary immunodeficiencies. Its immunomodulatory mechanisms include a decrease in regulatory T cells (Tregs) with the inhibition of Foxp3 expression, shifting the balance toward effector T cell activity. An increase in effector T cells (CD8+, Th1) with a boost in IFN-γ production enhances cytotoxic and helper T cell responses and an increase in Natural Killer (NK) cell activity, thus enhancing cytotoxicity via STAT4/STAT5 signaling [9].

It contributes to decreasing pro-inflammatory cytokines such as TNF-α, IL-6, and IL-1β and increasing, instead, anti-inflammatory ones with the suppression of the following key inflammatory pathways: NF-κB, MAPKs, JAK/STAT, and Notch-1. Moreover, curcumin is involved in the protection against sepsis-induced organ damage by the inhibition of inflammatory mediators (e.g., COX, iNOS, and AP-1) and a reduction in oxidative stress via PI3K/AKT and NF-κB pathway regulation. Furthermore, nanocurcumin and lipid nanoparticle formulations improve delivery, increase immune activation, and reduce inflammatory injury in sepsis models [10,11,12,13,14].

Despite the growing interest in natural compounds as adjuvants in cancer therapy, the potential of curcumin to enhance the efficacy and reduce the toxicity of conventional treatments in acute AML remains underexplored. This review aims to fill a critical gap by systematically evaluating preclinical and emerging clinical evidence on curcumin-based strategies, particularly in the context of nanoformulations, to improve therapeutic outcomes in AML patients. In fact, although several studies have investigated the anticancer properties of curcumin in solid tumors, its role in hematologic malignancies—especially AML—has received comparatively limited attention. Moreover, there is a lack of a comprehensive synthesis of how curcumin may modulate key molecular pathways involved in AML pathogenesis and treatment resistance. This review addresses this knowledge gap by integrating the current findings on curcumin’s pharmacodynamics, its synergistic potential with chemotherapeutic agents, and the advancements in delivery systems that may overcome its bioavailability limitations.

### 2.2. Curcumin and Acute Myeloid Leukemia

Numerous in vitro and vivo studies have highlighted the efficacy of curcumin against AML cells. Curcumin has shown antitumoral effects, both alone and when combined with other chemotherapeutic agents. It induces apoptosis in various AML cell lines through intrinsic and extrinsic mechanisms. These effects are mediated by mitochondrial membrane depolarization by increasing the inhibition of the B-cell lymphoma-XL protein, caspase activation, and the modulation of pro-apoptotic proteins (e.g., Bax) and anti-apoptotic proteins (e.g., Bcl-2). Furthermore, curcumin can inhibit the proliferation of leukemic cells and induce differentiation [15].

It also acts on the extrinsic apoptotic pathway by upregulating death receptors (DRs) on cells, triggering the tumor necrosis factor (TNF)-related apoptosis pathway and enhancing this process through the overexpression of DR4 and DR5 receptors [16,17].

**Figure 1:**
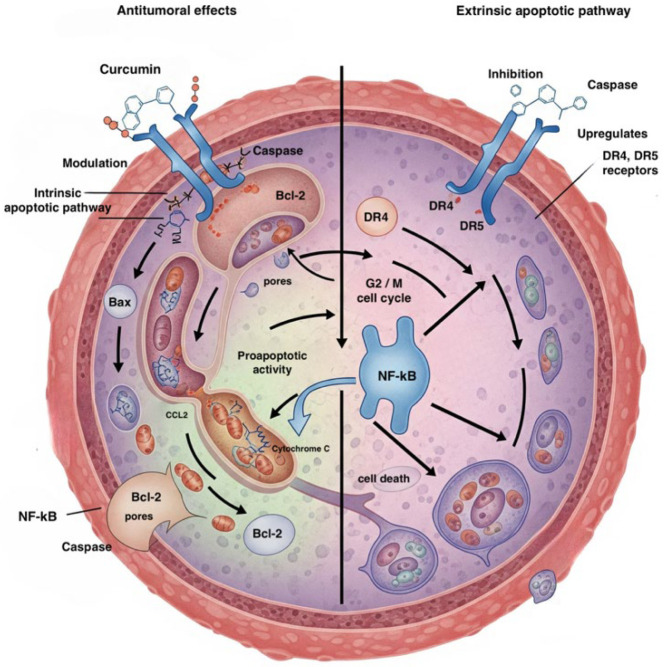
Pathways involved in the antitumoral effects of curcumin on an AML cell. Created in BioRender. Badagliacca, R. (2025). https://BioRender.com/78ibgti. Bcl-2 = B-cell lymphoma 2; Bax = Bcl-2-associated X protein; DR4 = Death Receptor 4; DR5 = Death Receptor 5; CCL2 = C-C motif chemokine ligand 2 (MCP-1); NF-kB = Nuclear Factor kappa-light-chain-enhancer of activated B cells. Refs. [15,19].

Apoptosis and cell cycle arrest are induced by the downregulation of NF-κB-regulated genes [18,19] like Bcl-2, Bcl-xL, cyclin D1, and cyclooxygenase-2 (COX-2) [20,21,22,23]. Curcumin is able to inhibit IκB kinase (IKK) and thus prevent the phosphorylation and subsequent degradation of the NF-κB inhibitor IκBα, blocking NF-κB activation, all of which leads to G2/M phase cell cycle arrest with apoptosis following [24] (Figure 1).

When cell cycle arrest happens and NF-kB is inhibited, curcumin can express its anti-proliferative effect by also suppressing the PI3K/AKT pathway, increasing the production of caspase-3 and BAX and decreasing BCL-2 levels [25]. By blocking PI3K/AKT, which is an important cellular pathway for several biological functions, growth, proliferation, and cellular metabolism are also compromised. On the other hand, the excessive activation of it leads to the proliferation of cancer cells with resistance also to chemotherapy.

**Figure 2:**
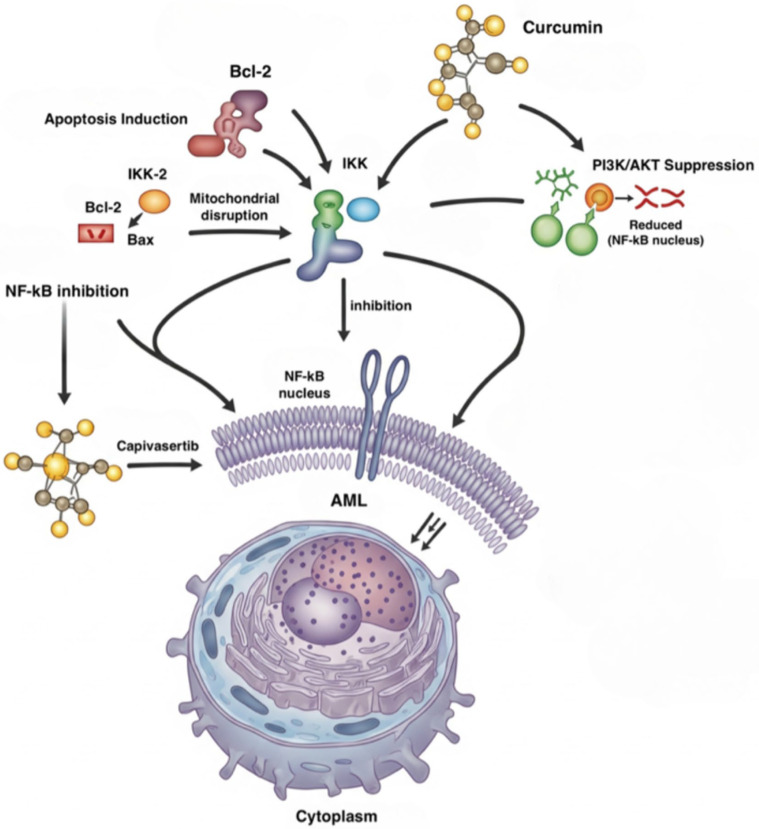
Curcumin and downregulation of the inflammatory signaling pathways. Created in BioRender. Badagliacca, R. (2025) https://BioRender.com/uqizprv. Bcl-2 = B-cell lymphoma 2; Bax = Bcl-2-associated X protein; NF-kB = Nuclear Factor kappa-light-chain-enhancer of activated B cells; IKK = inhibitor of nuclear factor-κB (IκB) kinase (IKK) complex; AML = acute myeloid leukemia; PI3K = Phosphoinositide 3-kinase; AKT = Protein kinase B (PKB) [25,26].

The suppression of inflammatory mediators led curcumin to be used also as a treatment in inflammatory diseases by the interaction between curcumin and toll-like receptors (TLRs) in order to control the activity of MAPK, NF-kB, and activator protein 1 (AP-1) [26]. MAPKs are important enzymes for the transduction of signals within cells and regulating cellular processes such as proliferation, differentiation, immune response, stress response, and even tumor progression. Furthermore, curcumin activates JNK and 25p38 MAPK in HL-60 acute myeloid leukemia cells, inducing caspase-dependent apoptosis [27] (Figure 2).

Another key role attributed to curcumin, as an antioxidant agent, is to neutralize the excess oxygen free radicals derived from oxidative stress, thus reducing reactive oxygen species (ROS) generation and increasing the efficacy of antioxidant enzymes [28,29].

Recently it has been demonstrated that ROS level is crucial in AML; curcumin reduces levels of ROS by downregulating pro-oxidant mediators such as NOX4 and improving antioxidant enzymes such as glutathione peroxidase [30,31].

Concerning the antitumoral context in which curcumin is also applied, angiogenesis, which is an important factor for growth and tumor proliferation, is inhibited by the polyphenol, blocking at the same time also interactions between the vascular endothelial growth factor (VEGF) and VEGFR2, suppressing the transcription of vascular growth factors mediated by NF-kB [32].

VEGF, a mediator of angiogenesis, is involved in the proliferation and progression of acute myeloid leukemia cells [33]; once the secretion of VEGF in LAM KG-1 and U937 cells is reduced by curcumin, it has been demonstrated that the anti-VEGF effects of the polyphenol, alone or, for instance, in combination with thalidomide, led to results that suggest the autocrine loop of VEGF as a promising therapeutic target able to influence the progression and development of AML cells.

Also, levels of osteopontin and CD44, mediators of adhesion and invasion, are reduced by curcumin. Furthermore, by the downregulation of MMP-2 and MMP-9, the remodeling of the extracellular matrix is limited. All these processes lead to the inhibition of metastases and tumor invasion [34].

Despite these encouraging preclinical findings, it is important to note that the vast majority of evidence for curcumin’s anti-leukemic effects comes from in vitro cell line studies and in vivo animal models. Differences in the metabolism, pharmacokinetics, and bioavailability between these models and humans limit the direct translation of these results into clinical practice. Therefore, well-designed clinical trials are urgently needed to determine whether curcumin’s efficacy and safety can be replicated in AML patients to establish optimal dosing and to evaluate potential interactions with standard chemotherapeutic agents.

## 3. Curcumin, Combination Therapies, and Synergistic Effects in Acute Myeloid Leukemia

Recent advances in cancer treatment aim to overcome multidrug resistance, boost effectiveness, and lessen side effects by combining anticancer drugs with other agents that have different targets or enhance drug sensitivity [35], as shown in Table 1 and Table 2. For instance, daunorubicin (DNR) has been paired with setanaxib, and venetoclax with hypomethylating agents for AML [36]. In addition, natural compounds such as curcumin are being combined with chemotherapies, such as doxorubicin. Doxorubicin is widely used also for leukemia and the combination with curcumin demonstrated a reduction in cardiotoxicity induced by doxorubicin. Combination is also able to induce a reduction in the development of multidrug resistance, by inhibiting drug efflux from cancer cells and enhancing doxorubicin absorption and intracellular retention [37].

Most of the patients with a tougher prognosis present AML cells that are CD34-positive. These CD34+ AML cells are also significantly more resistant to DNR, making them much harder to treat (about 10 to 15 times harder than CD34-negative cells). Some tough-to-treat leukemia cells (CD34+ AML) can survive chemotherapy drugs like DNR because they have elevated levels of a protein called Bcl-2. The anti-apoptotic protein Bcl-2 can contribute to CD34+ AML cell survival, and the downregulation of the Bcl-2 protein can show efficacy in treating DNR-insensitive CD34+ AML.

The ability of curcumin to reduce the expression of Bcl-2 that induces the apoptosis of daunorubicin-insensitive CD34+ AML cells lines has been demonstrated [38]. The main goal is to explore how well curcumin can kill these DNR-insensitive CD34+ AML cells. Researchers are testing it on specific CD34+ AML cell lines (KG1a, Kasumi-1), as well as on DNR-sensitive U937 AML cells and primary CD34+ AML cells taken directly from patients’ bone marrow. Previous research has shown that an abundance of the protein Bcl-2 can block DNR from inducing cell death in more mature U937 AML cells. Curcumin’s action has been observed among DNR-insensitive KG1a, Kasumi-1, and DNR-sensitive U937 cells through G1/S arrest and apoptosis via an intrinsic apoptotic pathway involving the downregulation of Bcl-2 protein, loss of MMP, and activation of caspase-3, followed by PARP degradation.

Thus, the combination therapy of curcumin with DNR decreases Bcl-2 expression and inhibits proliferation in a synergic way. This underlines curcumin’s ability to reduce DNR insensitivity by downregulating Bcl-2 in CD34+AML cell lines and in primary CD34+ AML cells, being a potential anti-leukemic agent for the treatment of DNR-insensitive CD34+ AML cells.

Improved apoptotic effects have also been found when combining curcumin with arsenic trioxide (ATO) and lonidamine. This happens through ROS, the activation of the mitochondrial pathway, and the dissipation of mitochondrial transmembrane potential. This also leads to enhancing the effectiveness of ATO and on myeloid leukemia cells [39,40].

Furthermore, by combining curcumin with valproic acid, a histone deacetylase inhibitor, it is possible to observe an increase in Sp1 binding and the acetylation of histones H3 and H4 in the Bax promoter region. This effect is able to cause the upregulation of Bax expression, the inhibition of proliferation, and cell death in acute promyelocytic leukemia (APL) HL-60 cells [41].

Moreover, involving thalidomide, an immunomodulatory agent with antitumor activity, and combining it with curcumin will lead to the downregulation of STAT3 and BCL-XL expression, thus inducing the inhibition of cell growth and apoptosis in AML cell lines [42,43]. TRAIL is a promising anticancer agent because it uniquely kills cancer cells while leaving healthy cells unharmed. TRAIL is a protein that triggers cell death in cancer cells by binding to specific “death receptors” (DR4 and DR5) on their surface.

Tumor necrosis factor-related apoptosis-inducing ligand (TRAIL/Apo2L) shows powerful anti-leukemic properties, although its clinical application has been blocked by the development of resistance to TRAIL-based monotherapies. Curcumin enhances TRAIL-induced apoptosis in various leukemic cell lines, including K562, MOLT4, HL60, and KG1, increases the expression of DR4 and DR5, and suppresses the FLICE-like inhibitory protein (cFLIP) and other anti-apoptotic proteins [44].

Furthermore, the combination of the IL2-TRAIL peptide and curcumin has shown cytotoxicity against peripheral blood mononuclear cells (PBMCs) from AML patients, showing an impressive 90% effectiveness [45]. Studies have shown that even low, non-toxic concentrations of curcumin can make TRAIL-resistant tumor cells vulnerable to cell death induced by TRAIL. For example, curcumin reduces levels of cell death-inhibiting proteins. Curcumin’s effect is partly due to its ability to develop ROS, which then upregulate DR5 receptors and activate TRAIL’s cell death pathway in various cancers [46,47]. It has been investigated how curcumin enhances the effectiveness of IL2-TRAIL and DR5 receptor-specific IL2-TRAIL peptide immunotoxins in leukemia. It has been found that pre-treating leukemic cells (from both cell lines and patient samples) with curcumin significantly increased their TRAIL death receptors, making them extremely sensitive to even low, non-lethal doses of TRAIL immunotoxins [48,49,50,51].

Scientific studies are also still ongoing with regard to the combination of curcumin with naringenin, which is a flavonoid extracted from citrus fruit, with potential anti-carcinogenic properties; this is the reason why studies aim to examine the influence of the two combined agents on THP-1 cells, assessing their effects on their progression through the cell cycle and their rate of programmed death.

So far, it has been revealed, through techniques like cell viability assays and flow cytometry, that naringenin boosted curcumin’s ability to kill cells and block their overall viability. Further investigation demonstrated that curcumin and naringenin together stopped the THP-1 cell cycle at both the S and G2/M phases [52].

In vitro studies have also examined the combination effects of curcumin with azacitidine (AZA). AZA is a drug that reduces methylation, working by blocking DNA methyltransferase. It is administered in low doses to avoid harming healthy cells. This allows AZA to reactivate tumor suppressor genes that cancer might have switched off [53].

In lab tests with leukemia cells, AZA has been shown to restore proper methylation, which in turn slows down cell growth, triggers programmed cell death, changes the cell’s life cycle, and encourages cells to develop normally.

Currently, both the FDA (Food and Drug Administration) and the EMA (European Medicines Agency) have approved this drug for treating myelodysplastic syndromes and for patients 65 years of age and older who have recently been diagnosed with AML [54,55,56]. The findings revealed a synergistic effect when AZA and curcumin were combined. Among all tested leukemia cell lines and most leukemia patient samples, a reduction in cell proliferation and increased apoptosis compared with drugs used alone was observed [57]. Importantly, the AZA and curcumin combination also demonstrated low toxicity to healthy cells.

These promising results are important for future research to investigate the clinical effectiveness of this drug combination.

A further therapeutic approach is investigated through the examination of the anti-leukemic effects of curcumin and carnosic acid (CA), another polyphenol combined with methyl 4-hydroxycinnamate (MHC) through in vitro and in vivo using AML cells [58,59].

Even at low doses, these combinations (MHC+CA and CUR+CA) significantly kill AML cells without affecting normal blood cells. Both combinations trigger cell death through apoptosis, which relies on calcium and caspase without an increase in harmful ROS.

Researchers believe that the shared ability of MHC+CA and CUR+CA to kill cells might be because MHC and CUR share structural features, specifically a 4-hydroxyl group on their aromatic ring and an α,β-unsaturated carbonyl group [60].

These findings also suggest that these synergistic combinations of natural or synthetic phenolic compounds could be extremely useful for developing new treatments or prevention strategies for AML.

Previous research has also indicated that curcumin may act synergistically with cytarabine (Ara-C), a common chemotherapy drug, in treating AML [61].

### Curcumin, Curcuminoid, and Chemoresistance

Curcumin diferuloylmethane is the most important curcuminoid with anti-inflammatory and antioxidant properties. Curcuminoids are a class of polyphenolic compounds primarily found in *Curcuma longa*, the plant from which turmeric is derived; several related compounds exist, with identical properties to curcumin.

Demethoxycurcumin is a curcumin variant with one less methoxy group and Bisdemethoxycurcumin lacks two methoxy groups compared with curcumin.

Recently, other synthetic and semi-synthetic derivates have been studied to improve some limitations of the therapeutic effectiveness of natural curcumin, such as chemical stability, pharmacological activity, and low oral bioavailability, since curcumin is rapidly metabolized and excreted. The main derivates are the following: Curcuminol, a hydroxylated derivative with enhanced antitumor activity; curcumin phosphate, which is useful in pharmaceutical formulations and is more water-soluble; EF24 is a synthetic analog with enhanced anticancer activity; and curcumin-β-cyclodextrin, which is useful for improving solubility and bioavailability.

Despite high rates of initial remission with chemotherapy, chemoresistance remains a significant clinical obstacle, leading to high relapse rates and poor patient outcomes [62]. Energy metabolism appears crucial in making AML cells, particularly resistant ones, more sensitive to Ara-C [63].

Involving xenograft tumors (created by injecting human AML cells into mice tails), the effects of various treatments have been investigated. These treatments included cytarabine, curcumin, VSL#3 (a probiotic mix), and terbinafine [64,65].

It was observed that curcumin enhanced the response to Ara-C in the xenograft model, but not directly in AML cells. In addition, this combination (curcumin with Ara-C) was shown to alter the gut microbiota; combining curcumin with Ara-C results in a distinct composition of microbiota compared with Ara-C treatment alone [66].

Another aspect to focus on is the action of tetrahydrocurcumin (THC). While curcumin primarily causes cell death through apoptosis, THC in HL60 cells mainly triggers cell death via autophagy [67].

Autophagy, through its forms of macroautophagy, microautophagy, and molecular chaperone, is a catabolic process that maintains cellular homeostasis and its purpose is cell survival during stress. The triggers of autopaghy are nutrient deprivation, stress, and hypoxia. The inhibition of autophagy has been studied as a potential strategy to make chemotherapy more effective, thus allowing cancer cells to overcome chemoresistance. On the other hand, apoptosis is a programmed cell death, induced by several cell signals, DNA damage, and toxins.

The question is whether curcumin and THC could induce these respective forms of cell death in HL60 cells that were resistant to Ara-C. The findings confirmed that curcumin initiated apoptosis and THC induced autophagy in the Ara-C-resistant HL60 cells; therefore both curcumin and THC could be valuable in treating AML that does not respond to Ara-C. Further details on THC reveal that it is a compound that lacks the α,β-unsaturated carbonyl group found in curcumin [68,69]. Despite this structural difference, its pharmacological activities are like curcumin’s. Other curcumin metabolites, such as curcumin glucuronide and curcumin sulfate, have been identified but are not considered bioactive [70].

With regard to the cytotoxicity in Ara-C-resistant AML cells, including HL60 (R-HL60) and AML cells taken directly from a patient, the findings of a scientific study showed that Ara-C, curcumin, and THC all induced cell death in these Ara-C-resistant AML primary cells. The patient-derived cells came from someone with relapsed AML who had not responded well to previous high-dose Ara-C chemotherapy. While the level of Ara-C resistance varied among individual cells both curcumin and THC consistently demonstrated effective cytotoxicity in the tested sample of Ara-C-resistant AML cells [71,72,73]. The dysregulation of the cellular processes of apoptosis and autophagy contributes to the development of cancer and its resistance to chemotherapy. However, curcumin and tetrahydrocannabinol show promise in overcoming this challenge because they have efficacy on cell lines that have become resistant to the chemotherapy drug Ara-C.

**Table 1:** In vitro studies.

Therapeutic Combination	Cell Model	Mechanism of Action	Main Outcome	References
Curcumin + DNR	CD34+ AML (cell lines and primary cells)	↓ Bcl-2 → ↑ sensitivity to DNR	Synergy in inhibiting proliferation and inducing apoptosis	[38]
Curcumin + ATO + lonidamine	Myeloid leukemia cells	↑ ROS, mitochondrial pathway activation, ΔΨm dissipation	Increased efficacy of ATO and lonidamine	[39]
Curcumin + ATO	CD34+ KG-1a	↑ Bax, ↓ Bcl-2, ↓ PARP	Increased apoptosis	[40]
Curcumin + valproic acid	HL-60 (APL)	↑ Sp1 binding, histone H3/H4 acetylation on Bax promoter	↑ Bax, ↓ proliferation, apoptosis	[41]
Curcumin + thalidomide	AML cell lines	↓ STAT3, ↓ BCL-XL, anti-angiogenesis	Growth inhibition, apoptosis	[42,43]
Curcumin + TRAIL/IL2-TRAIL peptide	K562, MOLT4, HL-60, KG-1, AML PBMCs	↑ DR4/DR5, ↓ cFLIP, ↑ ROS	90% efficacy on AML PBMCs, no toxicity to healthy cells	[44,45,46,47,48,49,50,51]
Curcumin + naringenin	THP-1	Cell cycle arrest at S and G2/M, ↑ apoptosis	Increased cytotoxicity vs. curcumin alone	[52]
Curcumin + AZA	AML cell lines and patient samples	↓ DNA methylation, reactivation of tumor suppressor genes	Synergy: ↓ proliferation, ↑ apoptosis, low toxicity to healthy cells	[53,54,55,56,57]
Curcumin + Ara-C	AML cells	—	No direct synergy in vitro	[61,62,63,64,65,66]
Curcumin + THC	HL60 Ara-C resistant cells	CUR → apoptosis, THC → autophagy	Effective also in Ara-C-resistant primary cells	[67,68,69,70,71,72,73]

Bcl-2 = B-cell lymphoma 2 protein; ROS = Reactive Oxygen Species; PARP = Poly(ADP-ribose) polymerase; APL = acute promyelocytic leukemia; Sp1 = Specificity protein 1; STAT3 = Signal Transducer and Activator of Transcription 3; TRAIL = Tumor necrosis factor-related apoptosis-inducing ligand; IL2 = Interleukin-2; DR = Death Receptor; PBMCs = Peripheral Blood Mononuclear Cells; cFLIP = Cellular FLICE-like inhibitory protein; AZA = Azacitidine; DNA = Deoxyribonucleic acid; Ara-C = Cytarabine; THC = Tetrahydrocurcumin.

**Table 2:** In vivo studies.

Therapeutic Combination	In Vivo Model	Mechanism/ Observations	Main Outcome	References
Curcumin + Ara-C	AML xenograft mouse model	Effect linked to gut microbiota and barrier integrity, not direct AML cell effect	Improved Ara-C response by strengthening intestinal barrier and reducing bacterial translocation	[64,65,66]
MHC + CA	AML in vivo	Ca^2+^-dependent apoptosis	Effective, no toxicity to healthy cells	[59]
Curcumin + CA	AML in vivo	Ca^2+^-dependent apoptosis without ROS	Selective cytotoxicity	[60]

Ara-C = Cytarabine; AML = Acute Myeloid Leukemia; MHC = Methyl 4-hydroxycinnamate; CA = Carnosic Acid; ROS = Reactive Oxygen Species.

## 4. Future Perspectives: Innovation and Nanotechnologies

Curcumin’s ability to improve the effectiveness of many chemotherapy drugs, such as doxorubicin, cisplatin, 5-FU, celecoxib, and paclitaxel, is achieved by making malignant cells more sensitive to chemotherapy, which can lead to lower clinical dosages and fewer side effects [37,74].

As already mentioned before, curcumin’s limitations are due to its instability, low absorption in the small intestine, and rapid elimination from the gallbladder; in fact, low levels of curcumin were revealed in serum and tissues through in vivo studies, even at high doses. In addition, it has a poor pharmacokinetic profile, rapid metabolism, limited water solubility, low oral bioavailability, and rapid systemic elimination [75,76]. Curcumin’s pharmacokinetic profile is markedly suboptimal, posing significant obstacles to its therapeutic application. Orally administered curcumin suffers from inefficient gastrointestinal absorption, primarily due to its pronounced hydrophobicity and extremely low solubility in aqueous environments (approximately 11 ng/mL at pH 5.0). Upon absorption, it undergoes rapid and extensive phase II metabolism in both hepatic and intestinal tissues, predominantly through glucuronidation and sulfation mediated by UDP-glucuronosyltransferases and sulfotransferases. These metabolic transformations yield conjugated derivatives—namely curcumin glucuronide and sulfate—that exhibit markedly diminished pharmacological activity. Additionally, curcumin is characterized by a short elimination half-life (generally less than one hour) and fails to achieve therapeutically relevant plasma concentrations, even when administered at high oral doses (up to 12 g/day), resulting in systemic bioavailability typically below 1%. To address these limitations, a variety of advanced drug delivery systems have been explored, all aimed at enhancing solubility, metabolic stability, and tissue targeting. Nonetheless, despite encouraging preclinical outcomes, the clinical translation of curcumin remains constrained by formulation scalability, inter-batch consistency, a lack of validated pharmacodynamic biomarkers, and regulatory complexities inherent to phytochemicals.

To overcome all these limitations, new drug delivery techniques have been explored with consequent better curcumin absorption and bioavailability. Among these are the use of curcumin analogs, nanoformulations, and incorporation into liposomes [77].

A significant role must be attributed to nanoformulation strategies, which are capable of improving problems related to the specific targeting of tumoral cells, biological activities, and solubility for the better transportation of insoluble drugs [78]. Liposomes play a key role as a drug delivery system in drug-resistant cancer therapy, reducing its toxicity. This nanotechnology-based drug delivery increases the bioavailability of unbound curcumin in plasma and improves its cellular uptake, absorption, permeability across the blood–brain barrier, and tissue dispersion. Moreover, plasma half-life is also increased and degradation in the small intestine is prevented [79]. In murine xenograft models, nanoformulations of curcumin, for example, liposomal curcumin at 50 mg/kg, reduced tumor burden by 40-50% and prolonged survival by 30% [80].

CD44 is a transmembrane glycoprotein expressed in AML and acts as a receptor for targeting drug release. It has been confirmed that new liposomes of curcumin express anti-leukemic properties because they have been modified with hyaluronan to target CD44 on AML cells [81]. Hyaluronan, as a target ligand, helps to deliver curcumin to CD44-overexpressing cells. This bond affinity for CD44 resulted in better efficacy in suppressing the proliferation of AML cells.

Focusing on delivery purposes, curcumin can also be encapsulated in micelles, vesicles made up of surfactant molecules with amphiphilic properties [82]. Curcumin micelles are used with efficacy for intravenous administration as aqueous formulations. Curcumin nanoparticles loaded with polylactic-co-glycolic acid (PLGA), a type of synthetic polymer conjugate, have shown enhanced bioavailability and triggered apoptosis in both in vitro and in vivo studies [83,84].

Doxorubicin is a widely used chemotherapy drug for various cancers, including leukemia. It works by interfering with DNA and RNA production [85,86,87,88]. While effective for many AML patients, leukemic stem cells (LSCs) are known to be less responsive to it [89,90]. Studies also explored a new drug delivery system using micelles to deliver Dox and curcumin, specifically to LSCs. The goal was to make the treatment more effective and less toxic for LSCs [91]. Dox–Cur micelles have been created (DCMs) to improve how cells absorb both drugs, and Cur micelles (CM) for comparison. To further target the LSCs, the micelles were modified with peptides that bind to the FLT3 receptor, a marker found on these cells.

Based on this study, the following gaps remain open: In vivo efficacy and safety, because no animal studies have yet explored this, biodistribution, pharmacokinetics (PK), pharmacodynamics (PD), in vivo targeting of bone marrow, stem cell niches, and in vivo toxicity. Additionally, the effect on relapse and leukemic stem cell eradication in vivo in order to understand if the dual-peptide Dox–Cur micelles can eliminate minimal residual disease and prevent AML relapse in models. Off-target effects and selectivity and how selective these micelles are for FLT3+ cells compared with normal hematopoietic stem/progenitor cells and possible toxicity in FLT3-expressing normal tissues. Mechanistic depth and an understanding of how curcumin + doxorubicin delivered in this way affect pathways of drug resistance, self-renewal, quiescence, cell signaling, and apoptosis in LSCs in more detail. Combination therapy and synergy and how these nanoparticulate micelle systems perform in combination with standard AML therapies (chemo, FLT3 inhibitors, etc.). Translational models and patient-derived cells, using patient-derived AML cells, primary LSCs, using PDX models and testing across AML with different FLT3 mutations/expression. Sample steps should be carried out, including pilot in vitro animal dosing studies and pilot biodistribution (fluorescent or other labeling) to confirm bone marrow targeting, with the survival benefit shown in AML xenografts or PDX models using dual-peptide DCM over a control and measuring LSC burden at around 5–6 months. Finally, the safety profile for the identification of dose-limiting toxicity; PK data (micelle comparison with free drug); after 12 months, the deployment of combination therapy experiments; and omics data to identify mechanisms and resistance remain research gaps.

A further therapeutic avenue involves the application of curcumin-conjugated nanobodies. However, the following key limitations should be improved: In vivo validation, since only in vitro cell lines have been tested and no animal model work to examine the pharmacokinetics, biodistribution, safety, and efficacy in a more realistic system (bone marrow niche, immune system) has been completed; bioavailability and stability in vivo, establishing curcumin’s metabolism and possible degradation, and determining how stable the particles are in circulation. The optimization of dosage, scheduling, and delivery route have to be defined, additionally, selectivity and off-target effects; though in vitro PBMC toxicity is low, in vivo there might be the expression of CD123 in other (normal) cell types, or possible immune reactions to the antibody, nanoparticles, etc. It has to be determined also mechanistic studies of how curcumin + anti-CD123 affects LSCs beyond apoptosis, for instance, effects on quiescence, self-renewal, signaling pathways, etc. The following specific objectives and milestones have to be carried out: Demonstrate that anti-CD123-Cur-NPs reduce leukemia burden by ≥50% versus non-targeted treatment in mouse xenograft models. Show that anti-CD123-Cur-NPs extend median survival in AML models by at least 30% over the control. Map signaling pathways modulated by treatment (e.g., reductions in stemness markers like CD34, FLT3, WT1, etc.) and show reduced colony formation in vitro by LSCs treated. Establish a dosing schedule that yields the effective suppression of AML with minimal toxicity to normal hematopoietic cells in vivo. Produce stable GMP-grade anti-CD123-Cur-NPs with reproducible properties in ≥3 independent batches; show stability at 4 °C and −20 °C for at least 6 months. Therefore, the expected impact will consist of a validated nanoparticle system that specifically targets leukemic stem cells in vivo, reducing AML disease burden and relapse risk. Moreover, a defined safety profile showing minimal off-target effects or toxicity, production methods ready for translation, with the potential to move toward early-phase clinical trials in AML (first in humans) with novel targeted therapy and a mechanistic understanding that supports biomarker-guided use.

Other open questions have to be improved: Longer-term in vivo studies and relapse prevention, because the in vivo work was short (2 weeks of treatment, follow-up unclear). Future studies need to determine whether disease relapse can be prevented and minimal residual disease eliminated, and the survival benefits over longer periods. In addition, combination therapy studies using HA-Cur-LPs together with standard of care AML treatments (chemotherapy, targeted agents, demethylating agents) to assess synergy or resistance and clinical relevance, as well as patient-derived models in order to use primary AML patient cells rather than cell lines. From a further detailed study, a complete biodistribution and PK pilot studies would be expected to determine half-life and primary organ accumulation.

Curcumin also helps reduce doxorubicin’s toxicity and may lessen drug resistance in AML cells and LSCs. The aim of DCMs is to target cancer cells, such as FLT3-positive AML-LSCs, through specific peptides on the surface [92,93,94]. Thus, peptide-conjugated curcumin micelles have an important role in increasing curcumin accumulation in FLT3-positive AML cells.

In conclusion, despite the promising preclinical outcomes, the clinical translation of nanoformulations remains challenging. Key hurdles include the scalability and reproducibility of nanoparticle synthesis, as many laboratory methods are difficult to translate to large-scale manufacturing while maintaining a consistent size, drug loading, and surface modifications. Manufacturing complexities also drive up production costs, limiting widespread clinical adoption. Regulatory hurdles are substantial because nanoformulations often behave differently than conventional small molecules, requiring extensive safety and pharmacokinetic evaluations. Novel toxicities have also been observed in preclinical studies, including unexpected organ accumulation and developmental toxicity in experimental animal models, raising concerns about long-term safety. Together, these factors underscore the need for robust quality control, detailed toxicological assessments, and strategic planning before nanoformulations can successfully progress to human clinical trials.

## 5. Conclusions

Nowadays, there are currently some ongoing studies on curcumin application in patients with other forms of acute and chronic leukemia such as children with acute lymphoblastic leukemia (ALL). The main goal of this study is assessing the biological effects of curcumin on microbiota by eliminating intestinal microflora dysbiosis, in the maintenance phase of chemotherapy through any reported adverse event [95]. Curcumin and cholecalciferol may prevent or slow the growth of cancer cells. A different study focused on the efficacy and tolerability of curcumin and cholecalciferol combination in treating patients with previously untreated stage 0-II chronic lymphocytic leukemia or small lymphocytic lymphoma. Patients achieving a partial response or better may receive treatment for a total of 2 years [96]. On the other hand, similar clinical studies on curcumin application in AML patients are currently lacking, highlighting a critical gap in translational research.

AML has the highest incidence and mortality rates among all leukemia categories. The relapse rate is still high, although a high percentage of complete remission is achieved with chemotherapy, leading to an unfavorable prognosis [97]. Nowadays, chemoresistance is still a challenge and curcumin demonstrated anti-proliferative properties and synergistic anti-leukemic effects with several targeted and conventional therapies in AML, primarily in preclinical models, as mentioned above.

It is important to emphasize that most evidence for curcumin’s efficacy in AML comes from in vitro experiments and animal studies. While these preclinical findings are promising, their direct translation to human patients is uncertain due to differences in metabolism, pharmacokinetics, and bioavailability. Bridging this translational gap requires well-designed clinical trials to confirm the safety, efficacy, and optimal dosing of curcumin in combination with conventional AML therapies.

Synergy has been shown with hypomethylating agents such as azacitidine, anthracyclines (doxorubicin, idarubicin), cytarabine, and arsenic trioxide, resulting in increased apoptosis, reduced proliferation, and, in animal models, improved survival compared with monotherapy.

Curcumin enhances apoptosis, cell cycle arrest, and chemosensitivity through the inhibition of key oncogenic pathways such as AKT, JAK2/STAT3, and FoxM1, and by modulating autophagy and immune surveillance.

Patients with AML, who take advantage of the combination of curcumin and targeted therapies, are those with relapsed or refractory disease and those exhibiting resistance to standard agents, such as cytarabine or anthracyclines. In addition, patients with CD44-positive AML may also benefit from curcumin formulations targeting CD44.

This evidence supports that curcumin also sensitizes AML cells to cytarabine and overcomes resistance, through the modulation of oxidative stress and the intestinal microbiota. Importantly, these effects are generally selective for leukemic cells, sparing normal hematopoietic progenitors [98]. Combination with arsenic trioxide or azacitidine augments anti-leukemic efficacy, including enhanced autophagy, immune activation (notably via the NKG2D-NKG2D-L axis), and the suppression of immune evasion [99,100].

Curcumin is generally well tolerated and has a wide therapeutic window. Furthermore, there are no established absolute contraindications to the use of curcumin in combination with standard therapies for AML. Preclinical studies demonstrate that curcumin, alone or in combination with these agents, does not increase cytotoxicity to normal hematopoietic cells. On the other hand, patients with pre-existing hepatic or biliary dysfunction and those on medications metabolized by cytochrome P450 enzymes should be monitored. Supervision is also necessary for those with compromised marrow or significant baseline cytopenias for potential myelosuppression.

In vitro and animal studies have not reported significant toxicity when curcumin is combined, for example, with arsenic trioxide, azacitidine, or anthracyclines, but clinical data are limited and do not exclude the possibility of adverse effects in humans.

Curcumin may potentiate the effects of chemotherapeutic agents by increasing ROS and apoptosis, which could theoretically enhance myelosuppression or other toxicities, though this has not been observed in preclinical models.

Nevertheless, patients receiving high-dose or nanoformulated curcumin may be at an increased risk for unanticipated toxicities due to improved bioavailability.

Nowadays, there is still no data on the pharmacokinetic interactions with standard AML therapies, but curcumin is known to interact with cytochrome P450 enzymes and thus could affect drug metabolism.

Despite promising available preclinical data, curcumin’s clinical application is currently limited by its poor bioavailability; therefore, additional broad clinical trials are needed to confirm efficacy in patients and improve curcumin’s absorption and stability.

The aim of ongoing trials is to better define the optimal dosage, the most effective combinations, and the reliable biomarkers that can predict which patients will respond well to it. Furthermore, other studies have been performed on Diarylheptanoids which play a pivotal role in mediating the therapeutic properties attributed to curcumins. These compounds constitute a distinct class of secondary metabolites characterized by a structural motif comprising two aromatic rings connected via a seven-carbon aliphatic chain. Predominantly found in species belonging to the *Curcuma* genus, diarylheptanoids have garnered considerable scientific attention due to their unique molecular architecture and extensive spectrum of biological activities. Notably, their anticancer potential has been well documented, underscoring their relevance in both medicinal chemistry and synthetic drug development [101].

Future perspectives aim to include curcumin in AML treatment guidelines, and so far, its use remains under investigation to achieve a safer profile. The American Society of Hematology and the National Comprehensive Cancer Network do not recommend curcumin or its derivatives as part of standard AML therapy because of a lack of demonstrative and effective clinical data in humans. Despite extensive research highlighting curcumin’s anti-inflammatory and anticancer properties, findings regarding its efficacy in hematological malignancies remain inconsistent. Although, several studies have demonstrated curcumin’s ability to induce apoptosis, inhibit proliferation, and modulate key signaling pathways in leukemia, lymphoma, and multiple myeloma cells, clinical outcomes vary significantly, with some trials reporting minimal impact on biomarkers. These discrepancies may stem from differences in curcumin formulations, bioavailability, treatment duration, and patient heterogeneity. Moreover, while curcumin has shown synergistic effects when combined with agents like lenalidomide, its standalone efficacy remains debated. Consequently, although curcumin holds promise as an adjunctive therapy, further rigorous, standardized clinical trials are essential to clarify its therapeutic role in hematological cancers.

While preclinical studies clearly demonstrate curcumin’s anti-leukemic effects, it is important to emphasize that these findings are largely limited to in vitro cell lines and in vivo animal models. Differences between these models and humans pose significant challenges for direct clinical translation. Therefore, well-designed clinical trials are urgently needed to determine whether the efficacy and safety observed in preclinical studies can be replicated in AML patients, to establish optimal dosing, evaluate potential drug interactions, and identify predictive biomarkers for patient selection.

## Data Availability

Not applicable.

## References

[1] Döhner H., Wei A.H., Appelbaum F.R., Craddock C., DiNardo C.D., Dombret H., Ebert B.L., Fenaux P., Godley L.A., Hasserjian R.P. (2022). Diagnosis and management of AML in adults: 2022 recommendations from an international expert panel on behalf of the ELN. Blood.

[2] Samarkhazan H.S., Zehtabcheh S., Rahmani Seraji H., Beqaj S.H., Tayefeh S., Mohammadi M.H., Aghaei M. (2025). Unveiling the potential of CLL-1: A promising target for AML therapy. Biomark. Res..

[3] Bhansali R.S., Pratz K.W., Lai C. (2023). Recent advances in targeted therapies in acute myeloid leukemia. J. Hematol. Oncol..

[4] Chen Y.F., Li J., Xu L.L., Găman M.A., Zou Z.Y. (2023). Allogeneic stem cell transplantation in the treatment of acute myeloid leukemia: An overview of obstacles and opportunities. World J. Clin. Cases.

[5] Liu J.M., Li M., Luo W., Sun H.B. (2021). Curcumin attenuates Adriamycin-resistance of acute myeloid leukemia by inhibiting the LncRNA HOTAIR/miR-20a-5p/WT1 axis. Lab. Investig..

[6] Urošević M., Nikolić L., Gajić I., Nikolić V., Dinić A., Miljković V. (2022). Curcumin: Biological Activities and Modern Pharmaceutical Forms. Antibiotics.

[7] Chota A., George B.P., Abrahamse H. (2021). Interactions of multi-domain pro-apoptotic and anti-apoptotic proteins in cancer cell death. Oncotarget.

[8] Kah G., Chandran R., Abrahamse H. (2023). Curcumin a Natural Phenol and Its Therapeutic Role in Cancer and Photodynamic Therapy: A Review. Pharmaceutics.

[9] Wang Y., Lu J., Jiang B., Guo J. (2020). The roles of curcumin in regulating the tumor immunosuppressive microenvironment. Oncol. Lett..

[10] Zoi V., Kyritsis A.P., Galani V., Lazari D., Sioka C., Voulgaris S., Alexiou G.A. (2024). The Role of Curcumin in Cancer: A Focus on the PI3K/Akt Pathway. Cancers.

[11] Vieira B.M., Ferreira Caetano M.A., de Carvalho M.T., Dos Santos Arruda F., Tomé F.D., Fernandes de Oliveira J., Soave D.F., Pereira J.X., Celes M.R.N. (2023). Impacts of Curcumin Treatment on Experimental Sepsis: A Systematic Review. Oxidative Med. Cell. Longev..

[12] Delshadi R., Bahrami A., McClements D.J., Moore M., Williams L. (2021). Development of nanoparticle-delivery systems for antiviral agents: A review. J. Control. Release.

[13] Dhar S., Bhattacharjee P. (2021). Promising role of curcumin against viral diseases emphasizing COVID-19 management: A review on the mechanistic insights with reference to host-pathogen interaction and immunomodulation. J. Funct. Foods.

[14] Allegra A., Mirabile G., Ettari R., Pioggia G., Gangemi S. (2022). The Impact of Curcumin on Immune Response: An Immunomodulatory Strategy to Treat Sepsis. Int. J. Mol. Sci..

[15] Entezari M., Tayari A., Paskeh M.D.A., Kheirabad S.K., Naeemi S., Taheriazam A., Dehghani H., Salimimoghadam S., Hashemi M., Mirzaei S. (2024). Curcumin in treatment of hematological cancers: Promises and challenges. J. Tradit. Complement. Med..

[16] Zhou H., Ning Y., Zeng G., Zhou C., Ding X. (2021). Curcumin promotes cell cycle arrest and apoptosis of acute myeloid leukemia cells by inactivating AKT. Oncol. Rep..

[17] Ashour A.A., Abdel-Aziz A.A.H., Mansour A.M., Alpay S.N., Huo L., Ozpolat B. (2014). Targeting elongation factor-2 kinase (eEF-2K) induces apoptosis in human pancreatic cancer cells. Apoptosis.

[18] Gachpazan M., Habbibirad S., Kashani H., Jamialahmadi T., Rahimi H.R., Sahebkar A. (2021). Targeting Nuclear Factor-Kappa B Signaling Pathway by Curcumin: Implications for the Treatment of Multiple Sclerosis. Adv. Exp. Med. Biol..

[19] Zamanian M.Y., Alsaab H.O., Golmohammadi M., Yumashev A., Jabba A.M., Abid M.K., Joshi A., Alawadi A.H., Jafer N.S., Kianifar F. (2024). NF-κB pathway as a molecular target for curcumin in diabetes mellitus treatment: Focusing on oxidative stress and inflammation. Cell Biochem. Funct..

[20] Bahadar N., Bahadar S., Sajid A., Wahid M., Ali G., Alghamdi A., Zada H., Khan T., Ullah S., Sun Q. (2024). Epigallocatechin gallate and curcumin inhibit Bcl-2: A pharmacophore and docking based approach against cancer. Breast Cancer Res..

[21] Singh S., Barnes C.A., D’Souza J.S., Hosur R.V., Mishra P. (2022). Curcumin, a potential initiator of apoptosis via direct interactions with Bcl-xL and Bid. Proteins.

[22] Amirsaadat S., Jafari-Gharabaghlou D., Dadashpour M., Zarghami N. (2023). Potential Anti-Proliferative Effect of Nano-Formulated Curcumin Through Modulating MicroRNA-132, Cyclin D1, and hTERT Gene Expression in Breast Cancer Cell Lines. J. Clust. Sci..

[23] Mohan M., Hussain M.A., Khan F.A., Anindya R. (2021). Symmetrical and un-symmetrical curcumin analogues as selective COX-1 and COX-2 inhibitor. Eur. J. Pharm. Sci..

[24] Rainey N.E., Moustapha A., Petit P.X. (2020). Curcumin, a Multi-Faceted Hormetic Agent, Mediates an Intricate Crosstalk between Mitochondrial Turnover, Autophagy, and Apoptosis. Oxidative Med. Cell. Longev..

[25] Yu C., Yang B., Najafi M. (2021). Targeting of cancer cell death mechanisms by curcumin: Implications to cancer therapy. Basic Clin. Pharmacol. Toxicol..

[26] Gao Y., Zhuang Z., Lu Y., Tao T., Zhou Y., Liu G., Wang H., Zhang D., Wu L., Dai H. (2019). Curcumin Mitigates Neuro-Inflammation by Modulating Microglia Polarization Through Inhibiting TLR4 Axis Signaling Pathway Following Experimental Subarachnoid Hemorrhage. Front. Neurosci..

[27] Hsiao P.C., Chang J.H., Lee W.J., Ku C.C., Tsai M.Y., Yang S.F., Chien M.H. (2020). The Curcumin Analogue, EF-24, Triggers p38 MAPK-Mediated Apoptotic Cell Death via Inducing PP2A-Modulated ERK Deactivation in Human Acute Myeloid Leukemia Cells. Cancers.

[28] Jakubczyk K., Drużga A., Katarzyna J., Skonieczna-Żydecka K. (2020). Antioxidant Potential of Curcumin- A Meta-Analysis of Randomized Clinical Trials. Antioxidants.

[29] Liao D., Shangguan D., Wu Y., Chen Y., Liu N., Tang J., Yao D., Shi Y. (2023). Curcumin protects against doxorubicin-induced oxidative stress by regulating the Keap1-Nrf2-ARE and autophagy signaling pathways. Psychopharmacology.

[30] Trombetti S., Cesaro E., Catapano R., Sessa R., Lo Bianco A., Izzo P., Grosso M. (2021). Oxidative Stress and ROS-Mediated Signaling in Leukemia: Novel Promising Perspectives to Eradicate Chemoresistant Cells in Myeloid Leukemia. Int. J. Mol. Sci..

[31] Valko M., Leibfritz D., Moncol J., Cronin M.T.D., Mazur M., Telser J. (2007). Free radicals and antioxidants in normal physiological functions and human disease. Int. J. Biochem. Cell Biol..

[32] Al-Ostoot F.H., Salah S., Khamees H.A., Khanum S.A. (2021). Tumor angiogenesis: Current challenges and therapeutic opportunities. Cancer Treat. Res. Commun..

[33] Salemi M., Mohammadi S., Ghavamzadeh A., Nikbakht M. (2017). Anti-Vascular Endothelial Growth Factor Targeting by Curcumin and Thalidomide in Acute Myeloid Leukemia Cells. Asian Pac. J. Cancer Prev..

[34] Younes M., Mardirossian R., Rizk L., Fazlian T., Khairallah J.P., Sleiman C., Naim H.Y., Rizk S. (2022). The Synergistic Effects of Curcumin and Chemotherapeutic Drugs in Inhibiting Metastatic, Invasive, and Proliferative Pathways. Plants.

[35] Broxterman H.J., Georgopapadakou N.H. (2005). Anticancer therapeutics: “Addictive” targets, multi-targeted drugs, new drug combinations. Drug Resist. Updates.

[36] Jonas B.A., Pollyea D.A. (2019). How we use venetoclax with hypomethylating agents for the treatment of newly diagnosed patients with acute myeloid leukemia. Leukemia.

[37] Tan B.L., Norhaizan M.E. (2019). Curcumin Combination Chemotherapy: The Implication and Efficacy in Cancer. Molecules.

[38] Rao J., Xu D.R., Zheng F.M., Long Z.J., Huang S.S., Wu X., Zhou W.H., Huang R.W., Liu Q. (2011). Curcumin reduces expression of Bcl-2, leading to apoptosis in daunorubicin-insensitive CD34+ acute myeloid leukemia cell lines and primary sorted CD34+ acute myeloid leukemia cells. J. Transl. Med..

[39] Sánchez Y., Simón G.P., Calviño E., de Blas E., Aller P. (2010). Curcumin stimulates reactive oxygen species production and potentiates apoptosis induction by the anti-tumor drugs arsenic trioxide and Lonidamine in human myeloid leukemia cell lines. J. Pharmacol. Exp. Ther..

[40] Fan J.X., Zeng Y.J., Wu J.W., Li Z.Q., Li Y.M., Zheng R., Weng G.Y., Guo K.Y. (2014). Synergistic killing effect of arsenic trioxide combined with Curcumin on KG1a cells. Zhongguo Shi Yan Xue Ye Xue Za Zhi.

[41] Chen J., Wang G., Wang L., Kang J., Wang J. (2010). Curcumin p38-dependently enhances the anti-cancer activity of valproic acid in human leukemia cells. Eur. J. Pharm. Sci..

[42] Mohammadi Kian M., Salemi M., Bahadoran M., Haghi A., Dashti N., Mohammadi S., Rostami S., Chahardouli B., Babakhani D., Nikbakht M. (2020). Curcumin Combined with Thalidomide Reduces Expression of STAT3 and Bcl-xL, Leading to Apoptosis in Acute Myeloid Leukemia Cell Lines. Drug Des. Dev. Ther..

[43] Shehzad A., Lee J., Lee Y.S. (2013). Curcumin in various cancers. BioFactors.

[44] Surapally S., Jayaprakasam M., Verma R.S. (2020). Curcumin augments therapeutic efficacy of TRAIL-based immunotoxins in leukemia. Pharmacol. Rep..

[45] Pesakhov S., Khanin M., Studzinski G.P., Danilenko M. (2010). Distinct combinatorial effects of the plant polyphenols curcumin, carnosic acid, and silibinin on proliferation and apoptosis in acute myeloid leukemia cells. Nutr. Cancer.

[46] Jung E.M., Lim J.H., Lee T.J., Park J.W., Choi K.S., Kwon T.K. (2005). Curcumin sensitizes tumor necrosis factor-related apoptosis-inducing ligand (TRAIL)-induced apoptosis through reactive oxygen species-mediated upregulation of death receptor 5 (DR5). Carcinogenesis.

[47] Park S., Cho D.H., Andera L., Suh N., Kim I. (2013). Curcumin enhances TRAIL-induced apoptosis of breast cancer cells by regulating apoptosis-related proteins. Mol. Cell. Biochem..

[48] Dai X., Zhang J., Arfuso F., Chinnathambi A., Zayed M.E., Alharbi S.A., Kumar A.P., Ahn K.S., Sethi G. (2015). Targeting TNF-related apoptosis-inducing ligand (TRAIL) receptor by natural products as a potential therapeutic approach for cancer therapy. Exp. Biol. Med..

[49] Pavan A.R., Bernardes da Silva G.D., Hartmann Jornada D., Chiba D., dos Santos Fernandes G.F., Chin C.M., dos Santos J.L. (2016). Unraveling the Anticancer Effect of Curcumin and Resveratrol. Nutrients.

[50] Madhumathi J., Sridevi S., Verma R.S. (2016). Novel TNF-related Apoptotic-inducing Ligand-based Immunotoxin for Therapeutic Targeting of CD25 Positive Leukemia. Target. Oncol..

[51] Madhumathi J., Sridevi S., Verma R.S. (2017). CD25 targeted therapy of chemotherapy resistant leukemic stem cells using DR5 specific TRAIL peptide. Stem Cell Res..

[52] Shi D., Xu Y., Du X., Chen X., Zhang X., Lou J., Li M., Zhuo J. (2015). Co-treatment of THP-1 cells with naringenin and curcumin induces cell cycle arrest and apoptosis via numerous pathways. Mol. Med. Rep..

[53] Martìn I., Navarro B., Solano C., Calabuig M., Hernández-Boluda J.C., Amat P., Remigia M.J., García F., Villamón E., Tormo M. (2019). Synergistic Antioncogenic Activity of Azacitidine and Curcumin in Myeloid Leukemia Cell Lines and Patient Samples. Anticancer Res..

[54] Su Y., Xu H., Xu Y., Yu J., Xian Y., Luo Q. (2012). Azacytidine inhibits the proliferation of human promyelocytic leukemia cells (HL60) by demethylation of MGMT, DAPK, and p16 genes. Hematology.

[55] Gambari R., del Senno L., Barbieri R., Viola L., Tripodi M., Raschellà G., Fantoni A. (1984). Human leukemia K-562 cells: Induction of erythroid differentiation by 5-azacytidine. Cell Differ..

[56] Nguyen A.N., Luna-Moran A., Richard N., Belka I., Brady H., MacBeth K.J. (2010). Azacitidine induces differentiation of acute myeloid leukemia cell lines along the granulocytic/monocytic lineage. Cancer Res..

[57] Hassan H.E., Keita J.A., Narayan L., Brady S.M., Frederick R., Carlson S., Glass K.C., Natesan S., Buttolph T., Fandy T.E. (2016). The combination of dimethoxycurcumin with a DNA methylation inhibitor enhances gene re-expression of promoter-methylated genes and antagonizes their cytotoxic effect. Epigenetics.

[58] Trachtenberg A., Muduli S., Sidoryk K., Cybulski M., Danilenko M. (2019). Synergistic Cytotoxicity of Methyl 4-Hydroxycinnamate and Carnosic Acid to Acute Myeloid Leukemia Cells via Calcium-Dependent Apoptosis Induction. Front. Pharmacol..

[59] Pesakhov S., Nachliely M., Barvish Z., Aqaqe N., Schwartzman B., Voronov E., Sharoni Y., Studzinski G.P., Fishman D., Danilenko M. (2016). Cancer-selective cytotoxic Ca2+ overload in acute myeloid leukemia cells and attenuation of disease progression in mice by synergistically acting polyphenols curcumin and carnosic acid. Oncotarget.

[60] Nelson K.M., Dahlin J.L., Bisson J., Graham J., Pauli G.F., Walters M.A. (2017). The Essential Medicinal Chemistry of Curcumin. J. Med. Chem..

[61] Liu J., Luo W., Chen Q., Chen X., Zhou G., Sun H. (2022). Curcumin sensitizes response to cytarabine in acute myeloid leukemia by regulating intestinal microbiota. Cancer Chemother. Pharmacol..

[62] Ferrara F., Schiffer C.A. (2013). Acute myeloid leukaemia in adults. Lancet.

[63] Cheng C., Yuan F., Chen X., Zhang W., Zhao X., Jiang Z., Zhou H., Zhou G., Cao S. (2021). Inhibition of Nrf2-mediated glucose metabolism by brusatol synergistically sensitizes acute myeloid leukemia to Ara-C. Biomed. Pharmacother..

[64] Saland E., Boutzen H., Castellano R., Pouyet L., Griessinger E., Larrue C., de Toni F., Scotland S., David M., Danet-Desnoyers G. (2015). A robust and rapid xenograft model to assess efficacy of chemotherapeutic agents for human acute myeloid leukemia. Blood Cancer J..

[65] Hosseini M., Rezvani H.R., Aroua N., Bosc C., Farge T., Saland E., Guyonnet-Dupérat V., Zaghdoudi S., Jarrou L., Larrue C. (2019). Targeting Myeloperoxidase Disrupts Mitochondrial Redox Balance and Overcomes Cytarabine Resistance in Human Acute Myeloid Leukemia. Cancer Res..

[66] Hueso T., Ekpe K., Mayeur C., Gatse A., Joncquel-Chevallier Curt M., Gricourt G., Rodriguez C., Burdet C., Ulmann G., Neut C. (2020). Impact and consequences of intensive chemotherapy on intestinal barrier and microbiota in acute myeloid leukemia: The role of mucosal strengthening. Gut Microbes.

[67] Tseng Y.H., Chiou S.S., Weng J.P., Lin P.C. (2019). Curcumin and tetrahydrocurcumin induce cell death in Ara-C-resistant acute myeloid leukemia. Phytother. Res..

[68] Wu J.C., Lai C.S., Badmaev V., Nagabhushanam K., Ho C.T., Pan M.H. (2011). Tetrahydrocurcumin, a major metabolite of curcumin, induced autophagic cell death through coordinative modulation of PI3K/Akt-mTOR and MAPK signaling pathways in human leukemia HL-60 cells. Mol. Nutr. Food Res..

[69] Wu J.C., Tsai M.L., Lai C.S., Wang Y.J., Ho C.T., Pan M.H. (2014). Chemopreventative effects of tetrahydrocurcumin on human diseases. Food Funct..

[70] Aggarwal B.B., Deb L., Prasad S. (2014). Curcumin differs from tetrahydrocurcumin for molecular targets, signaling pathways and cellular responses. Molecules.

[71] Chen L., Guo P., Zhang Y., Li X., Jia P., Tong J., Li J. (2017). Autophagy is an important event for low-dose cytarabine treatment in acute myeloid leukemia cells. Leuk. Res..

[72] De Vries J.F., Falkenburg J.H., Willemze R., Barge R.M.Y. (2006). The mechanisms of Ara-C-induced apoptosis of resting B-chronic lymphocytic leukemia cells. Haematologica.

[73] Pan S.T., Li Z.L., He Z., Qiu J., Zhou S. (2016). Molecular mechanisms for tumour resistance to chemotherapy. Clin. Exp. Pharmacol. Physiol..

[74] Bordoloi D., Kunnumakkara A.B. (2018). The Potential of Curcumin: A Multitargeting Agent in Cancer Cell Chemosensitization. Role Nutraceuticals Cancer Chemosensitizat..

[75] Zoi V., Galani V., Lianos G.D., Voulgaris S., Kyritsis A.P., Alexiou G.A. (2021). The Role of Curcumin in Cancer Treatment. Biomedicines.

[76] Bučević Popović V., Farhat E.K., Banjari I., Jeličić Kadić A., Puljak L. (2024). Bioavailability of Oral Curcumin in Systematic Reviews: A Methodological Study. Pharmaceuticals.

[77] Hegde M., Girisa S., Chetty B.B., Vishwa R., Kunnumakkara A.B. (2023). Curcumin Formulations for Better Bioavailability: What We Learned from Clinical Trials Thus Far?. ACS Omega.

[78] Tomeh M.A., Hadianamrei R., Zhao X. (2019). A Review of Curcumin and Its Derivatives as Anticancer Agents. Int. J. Mol. Sci..

[79] Bertoncini-Silva C., Vlad A., Ricciarelli R., Fassini P.G., Suen V.M.M., Zingg J. (2024). Enhancing the Bioavailability and Bioactivity of Curcumin for Disease Prevention and Treatment. Antioxidants.

[80] Nirachonkul W., Ogonoki S., Thumvijit T., Chiampanichayakul S., Panyajai P., Anuchapreeda S., Tima S., Chiampanichayakul S. (2021). CD123-Targeted Nano-Curcumin Molecule Enhances Cytotoxic Efficacy in Leukemic Stem Cells. Nanomaterials.

[81] Sun D., Zhou J.K., Zhao L., Zheng Z., Li J., Pu W., Liu S., Liu X., Liu S.J., Zheng Y. (2017). Novel Curcumin Liposome Modified with Hyaluronan Targeting CD44 Plays an Anti-Leukemic Role in Acute Myeloid Leukemia in Vitro and in Vivo. ACS Appl. Mater. Interfaces.

[82] Hafez Ghoran S., Calcaterra A., Abbasi M., Taktaz F., Nieselt K., Babaei E. (2022). Curcumin-Based Nanoformulations: A Promising Adjuvant towards Cancer Treatment. Molecules.

[83] Tima S., Anuchapreeda S., Ampasavate C., Berkland C., Okonogi S. (2017). Stable curcumin-loaded polymeric micellar formulation for enhancing cellular uptake and cytotoxicity to FLT3 overexpressing EoL-1 leukemic cells. Eur. J. Pharm. Biopharm..

[84] Panda A.K., Chakraborty D., Sarkar I., Khan T., Sa G. (2017). New insights into therapeutic activity and anticancer properties of curcumin. J. Exp. Pharmacol..

[85] Oyeyode M., Tempel M., Lakowski T.M., Davie J.R. (2025). DNA intercalating drugs: Mechanisms of action in cancer treatment. Adv. Biol. Regul..

[86] Hilmer S.N., Cogger V.C., Muller M., Le Couteur D.G. (2004). The hepatic pharmacokinetics of doxorubicin and liposomal doxorubicin. Drug Metab. Dispos..

[87] Gabizon A., Shmeeda H., Barenholz Y. (2003). Pharmacokinetics of pegylated liposomal Doxorubicin: Review of animal and human studies. Clin. Pharmacokinet..

[88] Minotti G., Menna P., Salvatorelli E., Cairo G., Gianni L. (2004). Anthracyclines: Molecular advances and pharmacologic developments in antitumor activity and cardiotoxicity. Pharmacol. Rev..

[89] Fernandez H.F., Sun Z., Yao X., Litzow M.R., Luger S., Paietta E., Racevskis J., Dewald G., Ketterling R., Bennett J. (2009). Anthracycline dose intensification in acute myeloid leukemia. N. Engl. J. Med..

[90] Tacar O., Sriamornsak P., Dass C.R. (2013). Doxorubicin: An update on anticancer molecular action, toxicity and novel drug delivery systems. J. Pharm. Pharmacol..

[91] Chueahongthong F., Chiampanichayakul S., Viriyaadhammaa N., Dejkriengkraikul P., Okonogi S., Berkland C., Anuchapreeda S. (2024). Cytotoxicity of Doxorubicin-Curcumin Nanoparticles Conjugated with Two Different Peptides (CKR and EVQ) against FLT3 Protein in Leukemic Stem Cells. Polymers.

[92] Sharma R.A., Gescher A.J., Steward W.P. (2005). Curcumin: The story so far. Eur. J. Cancer.

[93] Tima S., Okonogi S., Ampasavate C., Pickens C., Berkland C., Anuchapreeda S. (2016). Development and Characterization of FLT3-Specific Curcumin-Loaded Polymeric Micelles as a Drug Delivery System for Treating FLT3-Overexpressing Leukemic Cells. J. Pharm. Sci..

[94] Chueahongthong F., Tima S., Chiampanichayakul S., Dejkriengkraikul P., Okonogi S., Sasarom M., Rodwattanagul S., Berkland C., Anuchapreeda S. (2022). Doxorubicin-Loaded Polymeric Micelles Conjugated with CKR- and EVQ-FLT3 Peptides for Cytotoxicity in Leukemic Stem Cells. Pharmaceutics.

[95] Elsayed Ebeid F.S. (2024). Safety and Efficacy of Curcumin in Children with Acute Lymphoblastic Leukemia. ClinicalTrials.gov ID NCT05045443. NCT05045443.

[96] Caimi P. (2024). Curcumin and Cholecalciferol in Treating Patients with Previously Untreated Stage 0-II Chronic Lymphocytic Leukemia or Small Lymphocytic Lymphoma. ClinicalTrials.gov ID NCT02100423. NCT02100423.

[97] Papież M.A., Krzyściak W., Szade K., Bukowska-Strakovà K., Kozakowska M., Hajduk K., Bystrowska B., Dulak J., Jozkowicz A. (2016). Curcumin enhances the cytogenotoxic effect of etoposide in leukemia cells through induction of reactive oxygen species. Drug Des. Dev. Ther..

[98] Zhang J.R., Lu F., Lu T., Dong W., Li P., Liu N., Ma D., Ji C. (2014). Inactivation of FoxM1 transcription factor contributes to curcumin-induced inhibition of survival, angiogenesis, and chemosensitivity in acute myeloid leukemia cells. J. Mol. Med..

[99] Zeng Y.J., Liu F., Wu M., Wu X., Zhang D., Yuan Q., Zhou L., Wu Z. (2023). Curcumin combined with arsenic trioxide in the treatment of acute myeloid leukemia: Network pharmacology analysis and experimental validation. J. Cancer Res. Clin. Oncol..

[100] Zhang M., Chen S., Cui Y., Jiang J., Luo J., Gao Y., Zeng Y. (2025). Curcumin combined with arsenic trioxide enhances autophagy and immune surveillance to inhibit immune escape in acute myeloid leukemia. Int. Immunopharmacol..

[101] Sudarshan K., Yarlagadda S., Sengupta S. (2024). Recent Advances in the Synthesis of Diarylheptanoids. Chem. Asian J..

